# Crosstalk between Serum and Skin Sphingolipids in Psoriasis

**DOI:** 10.3390/ijms241914872

**Published:** 2023-10-03

**Authors:** Mateusz Matwiejuk, Hanna Myśliwiec, Bartlomiej Lukaszuk, Marta Lewoc, Hend Malla, Piotr Myśliwiec, Jacek Dadan, Adrian Chabowski, Iwona Flisiak

**Affiliations:** 1 Department of Dermatology and Venereology, Medical University of Bialystok, 15-540 Bialystok, Poland; mateusz.matwiejuk@sd.umb.edu.pl (M.M.);; 2 Department of Physiology, Medical University of Bialystok, 15-222 Bialystok, Poland; bartlomiej.lukaszuk@umb.edu.pl (B.L.);; 3 1st Clinical Department of General and Endocrine Surgery, Medical University of Bialystok, 15-276 Bialystok, Poland

**Keywords:** psoriasis, sphingosine-1-phosphate, sphinganine, sphingosine, sphinganine-1-phosphate, ceramide

## Abstract

Psoriasis is a chronic, complex, immunological disorder, which may lead to many different systemic complications. Sphingolipids, including ceramide, are bioactive lipids, which take part in the regulation of immune reactions, cell growth, and apoptosis. Twenty psoriatic patients and twenty-eight control subjects were included in the study. Skin (both lesional and non-lesional) and serum samples were collected from both the control group and the psoriatic patients. The levels of sphingosine (SFO), sphingosine-1-phosphate (S1P), sphingomyelin, sphinganine (SFA), sphinganine-1-phosphate (SFA1P), and ceramide (CER) were assessed in both tissue (t) and serum (s) samples using high-performance liquid chromatography (HPLC). We identified elevated serum levels of SFO, S1P, SFA, and SFA1P in psoriatic patients when compared to healthy individuals. As far as the lesional skin and serum of psoriatic patients are concerned, we demonstrated positive associations between CER_t and CER_s, SFA_t and CER_s, and SFO_t and CER_s. Additionally, we found negative correlations in the non-lesional skin and serum of psoriatic patients, including SFO_t vs. SFO_s, CER_t vs. SFA_s, CER_t vs. SFO_s, and SFO_t vs. SFA_s. Finally, we observed a positive correlation between S1P and SFA1P in both the serum samples of psoriatic patients and the serum samples of the control group. In this study, we did not observe any correlations between psoriasis area and severity index (PASI) scores and sphingolipid levels. In conclusion, our findings indicate an interplay between skin and serum lipids in psoriatic patients, which is not observed in healthy individuals.

## 1. Introduction

Psoriasis is a chronic, immune-mediated, non-contagious, multidisciplinary disorder that is widespread across the globe [1]. The estimated prevalence of psoriasis ranges from 0.27% to 11.4%, predominantly observed in the adult population [2]. Psoriasis has various subtypes, including inverse, plaque, erythrodermic, pustular, and guttate forms. The most prevalent subtype is plaque psoriasis, accounting for almost 90% of cases [3]. Plaque psoriasis is distinguished by well-defined, elevated, and erythematous skin lesions covered with silver scales [4]. The accelerated cell cycle of psoriatic keratinocytes and their resistance to apoptosis contributes to keratinocyte hyperproliferation, which is evident in histopathological examinations through the presence of parakeratosis and acanthosis [5].

Due to its inflammatory nature, psoriasis is not only associated with nail and joint involvement but can also coexist with a range of systemic conditions [6]. Some examples include the presence of metabolic syndrome, characterized by hypertension, hyperlipidemia, insulin resistance, and obesity. These factors, over time, can contribute to the development of conditions such as coronary artery disease, atherosclerosis, type 2 diabetes, and even myocardial infarction [7]. Psoriatic patients commonly experience abnormal lipid levels. Specifically, their serum levels of low-density lipoprotein (LDL) cholesterol, very low-density lipoprotein (VLDL) cholesterol, triglycerides, and total cholesterol are frequently elevated as compared to individuals without psoriasis. Concurrently, high-density lipoprotein (HDL) cholesterol tends to be diminished in individuals with psoriasis [8]. Despite the increasing number of studies investigating the potential mechanisms underlying psoriasis, the exact etiology of this disease remains unclear. Sphingolipids are one of the most important groups of human lipids [9]. Their common feature is a sphingoid core, which includes sphingosine. Precisely, the sphingoid basis is manufactured by the combination of a fatty acid (usually palmitate) and amino acid (mainly serine) [10]. Interestingly, sphingolipids constitute an exceptional subtype of lipids, capable of functioning both as structural components and signaling molecules [11]. Sphingolipids have diverse roles, including participation in cell growth, regulation of cell death, cell adhesion, modulation of immune activity, nutrient uptake, angiogenesis, inflammation, metabolism, autophagy, response to reactive oxygen species, and resistance against various stressors. Various types of sphingolipids exist, including sphingosine (SFO), sphingosine-1-phosphate (S1P), ceramides (CER), ceramide-1-phosphate (C1P), sphinganine (SFA), sphinganine-1-phosphate (SFA1P), sphingomyelin, galactosylceramide, glucosylceramide, and lactosylceramide [9]. Among the highly bioactive sphingolipids, S1P and CER stand out. These two distinct sphingolipids are associated with contrasting functions. CER primarily governs processes such as necrosis, apoptosis, stress responses, inflammation, and cell cycle arrest. In contrast, S1P primarily functions as a signaling lipid, regulating cell growth, migration, differentiation, and proliferation. Interestingly, S1P, when binding to cell surface receptors, can also initiate the process of angiogenesis [12]. A reduced concentration of CER in the skin is commonly associated with pathological skin symptoms, such as dryness and compromised skin barrier function. This phenomenon is often observed in such conditions as atopic dermatitis, psoriasis, and xerosis [13]. On the other hand, in different types of psoriasis, elevated levels of ceramides (C16:0, C18:0, C20:0, C22:0, C24:1), not only in plasma but also in the skin, were observed. Exceptionally, so far, C12:0-CER is the only sphingolipid that has been found to be decreased in non-lesional skin vs. lesional skin of psoriatic patients. The levels of hexosylceramide, or lactosylceramide, remain unchanged between psoriatic and non-psoriatic people. The degree of sphingomyelin is abnormal in psoriatic skin in a fatty acid chain length-dependent manner; specifically, the increase in C16:0, C24:1, and C24:0 sphingomyelins is marked [11].

In addition to their role in the pathogenesis of psoriasis, sphingolipids (mostly CER) can serve in human serum as indicators of metabolic disorders and atherosclerotic cardiovascular conditions. For instance, diabetic neuropathy is associated with higher plasma levels of C24 and C26 ceramides, and deoxy-C24 ceramide rates of C18:1 and C18:0 ceramides are also perceived as essential markers of cardiovascular events in healthy people. Moreover, an elevated level of C18:1 ceramide is regarded as an indicator of necrosis after coronary angiography procedures [9]. Furthermore, sphingomyelin and C24:1 are strongly connected with the cardiovascular death rate. Moreover, increased levels of C22:0 and C24:0 in plasma may indicate a slight improvement in verbal memory in response to exercise among patients with coronary artery disease, in whom these cognitive abilities are typically impaired. Furthermore, there is an inverse correlation observed between the serum levels of S1P and atherosclerotic disease. Additionally, deoxysphingolipids could potentially serve as biomarkers for the exacerbation of diabetes mellitus [9].

In recent years, researchers have predominantly focused on examining lipid levels in the serum and tissues of individuals with psoriasis. However, the precise connection between cutaneous and tissue sphingolipids in patients with psoriasis remains unclear. This present study aims to elucidate the relationship between bioactive sphingolipids in serum and psoriatic skin, in comparison to unaffected skin, in patients with psoriasis.

## 2. Results

### 2.1. Study Population

**Figure 1:**
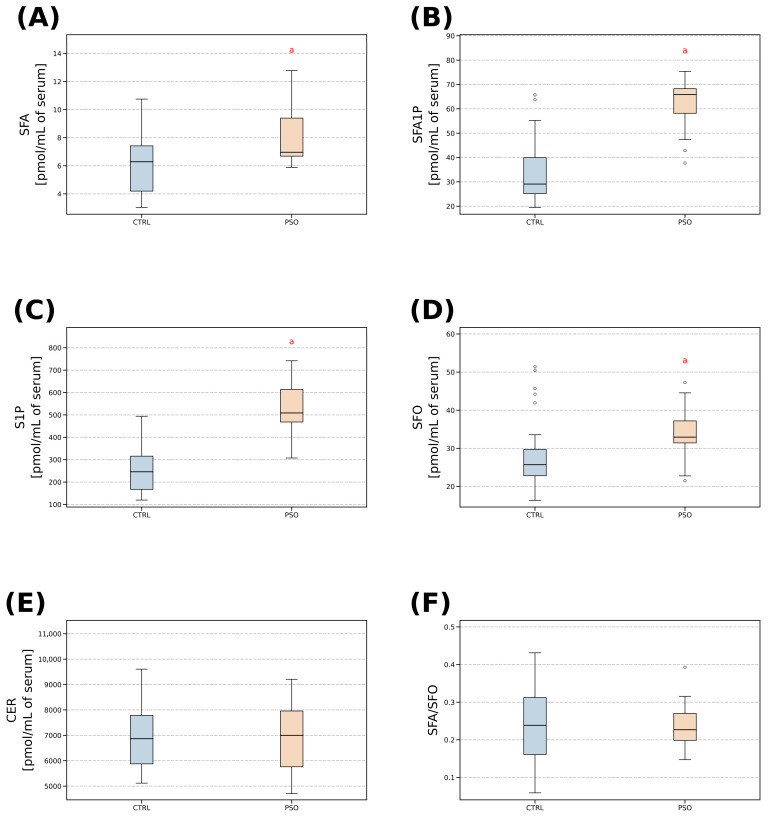
Comparison between sphingolipids in healthy (CTRL) and psoriatic (PSO) patients’ serum. (**A**) Amount of sphinganine (SFA) (pmol/mL of serum). (**B**) Amount of sphinganine-1-phosphate (SFA1P) (pmol/mL of serum). (**C**) Amount of sphingosine-1-phosphate (S1P) (pmol/mL of serum). (**D**) Amount of sphingosine (SFO) (pmol/mL of serum). (**E**) Amount of ceramide (CER) (pmol/mL of serum). (**F**) The ratio of sphinganine and sphingosine. Significance markers: “a” signifies different vs. CTRL (*p* < 0.05).

Twenty patients (13 males and 7 females) with active plaque-type psoriasis and twenty-eight subjects from the control group (8 males and 20 females) were included in the study. The mean age in the control group was 45.6 years, while in the psoriatic group, it was 53.2 years. The mean duration of psoriasis was 18.33 years, with a mean body mass of 87.2 kg, a mean height of 171.3 cm, and a median BMI of 29.9. The majority of psoriatic patients, *n* = 9, were overweight (45%), followed by *n* = 6 (30%) suffering from obesity, and *n* = 5 (25%) with a normal weight. Within the psoriatic group, 1 (5%) patient exhibited a mild form of psoriasis (PASI < 10), 13 (65%) individuals had a moderate form (PASI 10–20), and 6 (30%) suffered from severe psoriasis (PASI > 20). Table 1 presents the primary clinical characteristics of both the psoriatic and control groups. Our observations revealed statistically higher values (*p* < 0.05) of BMI, body mass, TAG, CRP, and TAG in psoriatic patients when compared to the control group. Table 2 presents concentrations of lipids measured in the psoriatic lesional skin, non-lesional psoriatic skin, skin of healthy subjects, and serum of psoriatic and healthy subjects. We observed statistically significant differences (*p* < 0.05) characterized by elevated levels of CER, S1P, SFA1P, SFA, and SFO in psoriatic lesional skin when compared to both psoriatic non-lesional skin and the skin of healthy subjects (Figure 1). Additionally, we found statistically significant variations (*p* < 0.05) featuring increased levels of CER and SFO in psoriatic non-lesional skin as compared to the skin of healthy subjects (Figure 1). Based on the serum sphingolipid concentrations, we observed a statistically significant difference (*p* < 0.05), characterized by elevated levels of S1P, SFA, and SFA1P, in the serum of psoriatic patients in contrast to the serum of healthy subjects (Figure 1). In this article, we have focused on the general amount of ceramides, without measuring the acyl chain composition.

### 2.2. Sphingolipid Parameters

It becomes evident that the concentrations of SFO, SFA, S1P, and SFA1P in the serum of patients with psoriasis were significantly elevated (*p* < 0.05) when compared to the levels of these sphingolipids in healthy individuals (Figure 1 and Table 1). While ceramide levels were found to be higher in psoriatic patients, the difference did not achieve statistical significance in comparison to healthy subjects.

**Figure 2:**
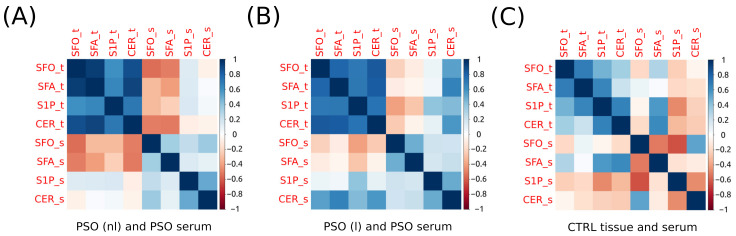
Correlation matrix (heatmap). Pearson correlation coefficients depicted as the shades of blue (positive correlation) or red (negative correlation). (**A**) Correlation matrix for psoriatic non-lesional (PSO (nl)) skin and serum. (**B**) Correlation matrix for psoriatic lesional (PSO (l)) skin and serum. (**C**) Correlation matrix for control (CTRL) skin and serum. CER—ceramide; S1P—sphingosine-1-phosphate; SFA—sphinganine; SFA1P—sphinganine-1-phosphate; SFO—sphingosine; suffix_s—serum; suffix_t—tissue.

We observed negative Pearson’s correlations between various variables in non-lesional psoriatic skin and the serum of psoriatic patients, all of which were statistically significant. Specifically, the correlations include SFO_t vs. SFO_s (*p* < 0.027), CER_t vs. SFA_s (*p* < 0.040), CER_t vs. SFO_s (*p* < 0.044), and SFO_t vs. SFA_s (*p* < 0.048) (Figure 2A).

A positive Pearson’s correlation was evident between several variables in lesional psoriatic skin and the serum of psoriatic patients. Specifically, statistically significant associations were observed between CER_t and CER_s (*p* < 0.026), SFA_t and CER_s (*p* < 0.006), and SFO_t and CER_s (*p* < 0.039) (Figure 2B).

There were no significant correlations between serum and skin sphingolipids in the healthy skin and serum of the control group (Figure 2C).

**Figure 3:**
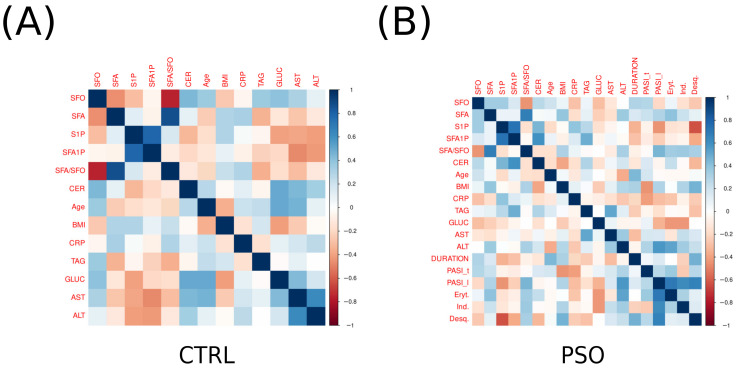
Correlation matrix (heatmap). Pearson correlation coefficients are depicted as shades of blue (positive correlation) or red (negative correlation). (**A**) Correlation matrix for non-psoriatic (CTRL) serum. (**B**) Correlation matrix for psoriatic (PSO) serum. ALT—alanine transaminase, AST—aspartate transaminase, BMI—body mass index, CER—ceramide, CRP—C-reactive protein, GLUC—glucose, S1P—sphingosine-1-phosphate, SFA—sphinganine, SFA1P—sphinganine-1-phosphate, SFO—sphingosine, TAG—triglycerides.

We identified significant positive Pearson’s correlations within the serum of both psoriatic patients and the control group, specifically between S1P and SFA1P. It is noteworthy that PASI scores did not exhibit significant correlations with sphingolipids in psoriatic serum. The correlations between erythema, induration, and desquamation with PASI, as well as SFA and SFO with SFA/SFO, were readily evident due to their calculated nature (Figure 3A,B).

## 3. Discussion

In this study, we aimed to elucidate the associations between sphingolipid levels in both healthy and psoriatic patients, taking into consideration clinical and laboratory data. We conducted an assessment and comparison of the concentrations of bioactive sphingolipids in both lesional and non-lesional skin, as well as serum samples from both healthy individuals and those with psoriasis. To the best of our knowledge, this comprehensive analysis has not been previously undertaken.

Our results reveal significant elevations in the serum levels of SFO, SFA, S1P, and SFA1P among psoriatic patients compared to the control group. In addition, the levels of ceramides were higher in patients with psoriasis, although this increase did not reach statistical significance when compared to healthy individuals.

### 3.1. The Role of Sphingosine-1-Phosphate

In our study, we identified elevated S1P levels in serum samples collected from patients with psoriasis when compared to the serum of healthy individuals (Figure 1). Looking at the function of S1P, it is a signaling molecule that plays an essential role in regulating angiogenesis, inflammation cascade, and vascular permeability [14]. In our study, we discovered a positive correlation between S1P and SFA1P in psoriatic serum (Figure 3).

Moreover, the raised plasma levels of S1P are noted not only in psoriasis but also in patients with obesity, in comparison to normal-weight people. It may be pointed out that S1P may have an expanded influence on various pathological disorders, and not only in psoriasis. Clearly, S1P has been well documented to correlate with metabolic irregularities, such as adiposity or insulin resistance. More precisely, there is a significant link between S1P and elements of metabolic syndrome; for example, fasting plasma insulin, total and LDL cholesterol levels, body fat percentage, and waist circumference are well described [15]. Furthermore, the aforementioned study revealed that psoriatic patients exhibited elevated serum concentrations of S1P, along with increased levels of serum alanine aminotransferase (ALT) and aspartate aminotransferase (AST), when compared to non-psoriatic patients [16]. These findings highlight a potential connection between the level of S1P, liver function, and metabolic disturbances in psoriatic patients.

S1P is well known for its ability to induce keratinocyte differentiation, exert antiproliferative and pro-inflammatory effects, and inhibit the growth of epidermal cells in mouse models of psoriasis, as demonstrated by Vaclavkova et al. [17]. Ponesimod is a selective modulator of sphingosine 1-phosphate receptor 1 (S1PR1). Its main role is blocking the process of the outflux of T cells from lymphoid organs. It is presumed that the inhibition of S1PR1 prevents the accumulation of pathogenic lymphocytes in both the skin and circulation, presenting a potentially successful approach for the treatment of psoriasis [17]. Moreover, D’Ambrosio et al. [18] reported promising results from phase II studies regarding the efficacy of ponesimod in relapsing–remitting psoriasis and also on multiple sclerosis. Their research indicates that lymphocyte reduction can establish the background for a selective S1PR1 modulator in multiple chronic and autoimmune diseases: for example, psoriasis. The quantity of peripheral blood T cells and B blood cells was reduced following the administration of 8 mg ponesimod. CD3+ and CD20+ counts were depleted significantly after the administration of 20–75 mg of ponesimod. Summing up, this paper suggests that ponesimod may lower the inflammatory cells, consequently reducing the inflammation process in psoriasis, which is of course one of the backgrounds of this skin disease [18].

### 3.2. Characteristics of Sphingosine and Sphinganine

In our study, we have made an intriguing observation of significantly elevated concentrations of both SFA and SFO in psoriatic serum, in comparison to healthy serum (Figure 1). We spotted a negative Pearson’s correlation between SFO_t vs. SFO_s (*p* < 0.027), CER_t vs. SFA_s (*p* < 0.040), CER_t vs. SFO_s (*p* < 0.044), and SFO_t vs. SFA_s (*p* < 0.048) in non-lesional psoriatic skin and the serum of psoriatic patients, which were all statistically significant (*p* < 0.05). Moreover, we describe positive Pearson’s correlations between SFA_t vs. CER_s (*p* < 0.006) and SFO_t vs. CER_s (*p* < 0.039) in lesional psoriatic skin and the serum of psoriatic patients, which are all statistically significant. The abovementioned correlations are presented in Figure 2.

The aforementioned results, detailing lipid abnormalities present in patients with psoriasis, emphasize the potential correlation between skin and serum lipids in the context of psoriasis and may elucidate the systemic involvement of sphingolipids and their interactions in this disease.

SFA is manufactured through the enzymatic combination of serine and palmitoyl-CoA by an enzyme called serine palmityltransferase. Afterwards, SFA is acylated to the CER. Subsequently, it is metabolized into glucosylceramide or sphingomyelin and may be next transformed into SFO and fatty acids via the action of an enzyme called ceramidase [19]. This enzymatic pathway is essential in the regulation and metabolism of sphingolipids in the human body. However, the relatively modest proportion (5–6% of total lipids) of these sphingoid bases, namely SFO and SFA, within the stratum corneum, holds significant importance in cell differentiation and proliferation, the preservation of lipid lamellae integrity, and antimicrobial protection.

Previous studies investigating the composition of SFO and SFA in psoriatic skin are limited. Regarding the changes in SFO and SFA in psoriatic serum, we are the first authors to describe these alterations. However, our scores are compatible with the findings presented by Sung-Hyuk et al. [19], who noticed an elevated amount of SFO and SFA, but only in psoriatic skin. Furthermore, Sorokin et al. [20] also show similar results regarding SFA quantity in the skin of psoriatic patients. These consistent findings across limited research provide strong evidence for the involvement of SFO and SFA in the pathogenesis of psoriasis. This underscores their potential significance as indicators or targets for therapeutic interventions in psoriasis [20].

Unfortunately, there are still no relevant data presenting the changes in SFA and SFO in serum of skin disorders other than psoriasis. Similar to psoriasis, the sphingolipid composition of the skin in atopic dermatitis (AD) has also been well studied. Toncic et al. [21] showed that the amounts of SFO and SFA soar in AD versus in healthy skin. However, no similar disturbances were described in non-lesioned atopic skin. Interestingly, our previous study revealed a similar elevation of SFO and SFA in psoriatic lesions, but we additionally observed an increase in SFO levels in non-lesioned psoriatic skin [22]. In AD, contrary to our new results, the ratio of SFA/SFO is reduced in healthy skin by comparison to non-lesioned and lesioned AD skin, which is reported by Toncic et al. [21]. Summing up, these outcomes underline the remarkable sphingolipid alterations in various skin disorders and still highlight the tangled role of sphingolipids in the pathogenesis of AD and psoriasis.

### 3.3. The Characteristics of Ceramides

However, in this study, we did not find any significant differences in CER concentration in psoriatic serum samples compared to healthy individuals (Figure 1). Interestingly, we observed a positive Pearson’s correlation between CER_t vs. CER_s (*p* < 0.026), SFA_t vs. CER_s (*p* < 0.006), and SFO_t vs. CER_s (*p* < 0.039) in psoriatic lesional skin and the serum of psoriatic patients. Additionally, we noted a negative Pearson correlation between CER_t vs. SFA_s (*p* < 0.040) and CER_t vs. SFO_s (*p* < 0.044) in non-lesional psoriatic skin and the serum of psoriatic patients. These correlations are presented in Figure 2.

Tawada et al. [23] reported that CER quantities depend on constant sustainability between their manufacture formation and degradation. The main cause of the abnormal production of CER in psoriasis is the reduced activity of ceramide synthase [23]. Alessandrini et al. [24] presented that disturbed CER amounts can be also decreased due to a decrease in sphingomyelinase activity, which is another crucial enzyme, in addition to ceramide synthase, involved in CER synthesis. Subsequently, these ceramide levels may be reduced due to the amount of prosaposin, which is a saposin precursor and plays a major role in the process of the hydrolysis of sphingolipids [24]. According to Lew et al. [13], in psoriasis, the amount of ceramide in the epidermis is depleted together with c-Jun N-terminal kinase and the protein kinase C alpha. Overall, the lower CER can result in the downregulation of these crucial kinases, which are known for their apoptotic cell signaling features. Consequently, this may lead to a reduction in sphingomyelinase-induced ceramides and at the same time a boost in the anti-apoptotic and proliferative characteristics of the psoriatic epidermis. [13].

In another study conducted on samples of psoriatic skin by Checa et al. [11], the authors demonstrated an elevated amount of CER in the skin of psoriatic patients compared to healthy individuals. Their outcome is consistent with that of our previous study, Matwiejuk et al. [22]. However, in contrast, Cho et al. [25] reported that the rate of CER in the lesional epidermis of psoriatic patients was more decreased than in the unlesioned epidermis [25]. The lack of significant differences in serum ceramide (CER) levels between psoriatic patients and healthy samples in our current study may be attributed to several factors. One critical consideration is the potential influence of various factors, such as differences in sample collection methods, variations in disease severity, and diverse patient characteristics, all of which could contribute to these similar outcomes. Notably, it should be highlighted that Cho et al. [25] conducted their investigation with a distinct patient cohort (10 patients), which is in contrast with our larger sample size (20 patients). This discrepancy in the number of individuals examined might provide a more comprehensive and accurate insight compared to the study of Cho et al. [25], given our broader representation of psoriasis-affected patients. Additionally, studies on greater numbers of patients still need to be performed. Even for patients suffering from AD, which was also described as disturbed amounts of ceramides, the alteration of ceramides was noted by Toncic et al. [21], who reported higher quantities of CER and glucosylceramide. Of course, these impairments are more marked in psoriatic lesions, but significantly different levels were also noted in non-lesional skin. In our previous study on psoriatic patients, Matwiejuk et al. [22] described similar observations where the CER level was higher in the lesioned skin compared to non-lesional psoriatic skin samples and healthy skin. However, in the study carried out on serum, we did not discover elevated levels of CER in healthy patients versus psoriatic patients. These similar results between the quantity of CER in the skin and serum point out that changes in CER metabolism may be similar in inflammatory skin diseases such as AD and psoriasis. Secondly, the abnormality of CER amounts may contribute to the disturbed skin barrier and cause the inflammatory processes. Still, subsequent studies are needed to discover how specific mechanisms underly CER metabolism in various skin diseases and the implications for disease pathogenesis and potential modalities.

The relationship between ceramides in the skin tissue and their influence on serum ceramide levels is not yet fully understood. In the present study, we found different significant and negative correlations between CER_t vs. SFA_s (*p* < 0.040) and CER_t vs. SFO_s (*p* < 0.044) in non-lesional psoriatic skin and the serum of psoriatic patients. Moreover, in lesional psoriatic skin and the serum of psoriatic patients, we noted a positive Pearson’s correlation between SFA_t vs. CER_s (*p* < 0.006), CER_t vs. CER_s (*p* < 0.026), SFO_t vs. CER_s (*p* < 0.039). These correlations are presented in Figure 2. These findings may point out that there are various connections between different sphingolipids in psoriasis which may be the background pathomechanism of psoriasis. However, it remains unclear how alterations in skin ceramide metabolism may contribute to alterations in ceramide quantities in the serum of psoriatic patients. Moreover, CER is engaged in many comorbidities other than psoriasis and AD, including components of metabolic syndrome. Hao et al. [26] stated that a metabolic syndrome is a group of abnormalities that enhance the chance of cardiovascular disease and diabetes [26]. Lee et al. [27] showed that CER is linked with insulin resistance (IR), obesity, dyslipidemia, and other metabolic abnormalities [27]. The relationship between skin and serum ceramides is complex and intricate. While alterations in ceramide composition have been observed in both lesional and non-lesional skin of patients with various dermatological conditions, the precise cause-and-effect dynamics between skin ceramides and serum ceramides remain elusive. Further research is needed to elucidate the potential bidirectional influence of these compartments on each other, shedding light on their intricate interconnections and implications for skin health and systemic conditions.

We must acknowledge that our study has some limitations and does not offer insights into the acyl chain composition within ceramides, nor provide data on the levels of enzymes engaged in sphingolipid biosynthesis. Addressing these limitations in future studies could contribute to a more comprehensive understanding of the sphingolipid dynamics in the context of the studied conditions. Furthermore, conducting research with a larger cohort of patients could help establish the significance and severity of sphingolipid abnormalities in the context of psoriasis. While our current findings provide valuable insights into sphingolipid disturbances and their interplay between psoriatic serum and skin, expanding the sample size would enhance the statistical power and enable us to draw more robust conclusions regarding the involvement of CER in psoriasis pathogenesis.

## 4. Materials and Methods

Twenty patients (13 male and 7 female) with active plaque-type psoriasis, with a mean age of 53.2, and twenty-eight healthy controls (8 male and 20 female), with a mean age of 45.6, were enrolled in the study. The severity of psoriasis was estimated using the Psoriasis Area and Severity Index (PASI) [28]. Body mass index (BMI) was calculated based on self-reported weight and height. None of the patients or controls were under dietary restriction. History of hypertension, liver disease (e.g., non-alcoholic fatty liver disease (NAFLD)), heart disease, and diabetes mellitus, and the results of the laboratory tests were collected from the hospital records of the patients. Laboratory tests were measured before treatment. All psoriatic patients gave their written informed consent before enrolment in the study. The study protocol was approved by the local university bioethical committee (no APK.002.500.2021), according to the principles of the Helsinki Declaration. Peripheral blood samples were taken before starting the treatment after overnight fasting. After centrifugation, the serum was stored at −80 °C until it was analyzed. Three mm punch biopsies were obtained from both the non-lesional and lesional skin from the trunk of psoriatic patients following local anesthesia. Samples from healthy patients were collected from the wound’s edge during a planned inguinal hernia operation under general anesthesia. Furthermore, in line with the PASI score, we assessed the clinical features of erythema, induration, and desquamation within the chosen psoriatic lesion for biopsy. The scoring system ranged from 0 to 4.

### 4.1. Sphingolipid Analysis

The contents of sphingosine, sphinganine, sphingosine-1-phosphate (S1P), and SFA1P were measured simultaneously using the method of Min et al. (2002) [29]. Briefly, tissues were homogenized in a solution composed of 25 mM HCl and 1 mM NaCl. Acidified methanol and internal standards (10 pmol of C17-sphingosine and 30 pmol of C17-sphingosine-1-phosphate; Avanti Polar Lipids (Alabaster, AL, USA)) were added and the samples were ultrasonicated in ice-cold water for 1 min. Lipids were then extracted by the addition of chloroform, 1 M NaCl, and 3 N NaOH. The alkaline aqueous phase containing S1P and SFA1P was transferred to a fresh tube. The residual phosphorylated sphingoid bases in the chloroform phase were re-extracted twice with methanol/1 M NaCl (1:1, *v*/*v*) solution and then all the aqueous fractions were combined. The amount of S1P and SFA1P was determined indirectly after dephosphorylation to sphingosine and sphinganine, respectively, with the use of alkaline phosphatase (bovine intestinal mucosa; Fluka (Seelze, Germany)). To improve the extraction yield of released sphingosine and sphinganine, some chloroform was carefully placed at the bottom of the reaction tubes. The chloroform fractions containing free sphingosine and sphinganine or dephosphorylated sphingoid bases were washed three times with alkaline water (pH adjusted to 10.0 with ammonium hydroxide) and then evaporated under a nitrogen stream. The dried lipid residues were redissolved in ethanol, converted to their o-phthalaldehyde derivatives, and analyzed using a high-performance liquid chromatography (HPLC) system (PROSTAR; Varian Inc. (Palo Alto, CA, USA)) equipped with a fluorescence detector and C18 reversed-phase column (OMNISPHER 5, 4.6 × 150 mm; Varian Inc.). The isocratic eluent composition of acetonitrile: water (9:1, *v*/*v*) (Merck, Darmstadt, Germany) and a flow rate of 1 mL min^−1^ were used.

The content of ceramide was determined by the procedure previously described by Baranowski et al. [30]. A small volume of the chloroform phase containing lipids extracted as described above was transferred to a fresh tube containing 31 pmol of C17-sphingosine as an internal standard. The samples were evaporated under a nitrogen stream, redissolved in 1 M KOH in 90% methanol, and heated at 90 °C for 60 min to convert ceramide into sphingosine. This digestion procedure does not convert complex sphingolipids, such as sphingomyelin, galactosylceramide, or glucosylceramide, into free sphingoid bases [31]. Samples were then partitioned by the addition of chloroform and water. The upper phase was discarded and the lower phase was evaporated under nitrogen. The content of free sphingosine liberated from ceramide was then analyzed using HPLC as described above. The calibration curve was prepared using N-palmitoylsphingosine (Avanti Polar Lipids) as a standard. The chloroform extract used for the analysis of ceramide level also contained small amounts of free sphingoid bases. Therefore, the content of ceramide was corrected for the level of free sphingosine determined in the same sample.

### 4.2. Statistical Analysis

The obtained data were analyzed using the R (ver. 4.2.2) statistical package. At the onset of analyses, the continuous data were checked for normality (Shapiro–Wilk test) and homoscedasticity (Fligner–Killeen test). Based on the above, between-group comparisons were made using parametric (ANOVA with subsequent pairwise Student’s *t*-test) or nonparametric methods (Kruskal–Wallis test with subsequent pairwise Wilcoxon test). The obtained *p*-values were adjusted for multiple comparisons (Benjamini–Hochberg correction). The corrected *p*-values lower than 0.05 were considered to be statistically significant. The results were presented in the form of box–whisker plots. The dependence between the variables of interest was determined based on Pearson’s coefficients and depicted using heatmaps.

## 5. Conclusions

The observed differences in the sphingolipid profile and the correlations between bioactive sphingolipids in psoriatic skin and serum, as well as healthy skin and serum, demonstrate the significant contributions of these lipids to one of the many pathways involved in the pathogenesis of psoriasis. These varieties, which are more strongly highlighted in psoriatic skin and serum, underline the potential basis mentioned earlier in this article. Moreover, the engagement in the inflammatory process links this etiology of psoriasis with other diseases such as metabolic syndrome and AD.

Furthermore, the finding that sphingolipid metabolism is impaired not only in the affected skin but also in serum, at the same time, suggests a broader systemic involvement and various potential correlations between specific types of sphingolipids. These implications can lead to lipid metabolism abnormalities, which can be extended further than the visible psoriatic lesions and might play an important role in the etiology of this disease. Further studies are necessary to clarify the clinical importance of lipid abnormalities in psoriasis and their potential usefulness as therapeutic targets.

## Figures and Tables

**Table 1:** Clinical and biochemical characteristics of the control group (CTRL) and psoriatic patients (PSO).

Variables	CTRL	PSO
**Age (years)**	45.6 ± 15.14	53.2 ± 15.66
**Body mass (kg)**	73.8 ± 9.47	87.2 ± 15.95 a
**Height (cm)**	171.2 ± 7.17	171.3 ± 8.6
**BMI (kg/m2)**	25.18 ± 2.84	29.9 ± 6.49 a
**CRP (mg/dL)**	1.4 ± 0.83	8.2 ± 12.44 a
**Glucose (mg/dL)**	97.1 ± 16.58	89.1 ± 17.4
**TAG (mg/dL)**	81.9 ± 35.83	116.35 ± 40.89 a
**AST (U/L)**	23.3 ± 8.53	24.1 ± 12.42
**ALT (U/L)**	21.1 ± 16.09	22.8 ± 12.4
**Sex (no. female/no. male)**	20/8	7/13 a

Data are presented as the mean ± standard deviation. a—different vs. PSO (*p* < 0.05); BMI—body mass index, CRP—C reactive protein, TAG—triglycerides, AST—aspartate transaminase, ALT—alanine transaminase.

**Table 2:** Concentration of sphingolipids in the skin (pmol/mg of tissue, mean (standard deviation)) and serum (pmol/mL of serum, mean (standard deviation)).

Sphingolipid	CTRL_t	PSO_t (nl)	PSO_t (l)	CTRL_s	PSO_s
**CER**	7.82 (3.62)	24.96 (13.69) a	69.19 (28.71) ab	6920.52 (1254.24)	6882.53 (1284.32)
**S1P**	0.15 (0.04)	0.18 (0.07)	0.41 (0.18) ab	252.41 (100.04)	528.77 (121.23) a
**SFA1P**	0.03 (0.01)	0.03 (0.01)	0.07 (0.03) ab	33.59 (12.81)	62.13 (10.95) a
**SFA**	0.16 (0.04)	0.23 (0.17)	0.68 (0.43) ab	6.13 (2.02)	7.83 (1.87) a
**SFO**	0.29 (0.09)	0.6 (0.53) a	1.9 (0.84) ab	28.55 (9.49)	33.53 (6.54)
**SFA/SFO**	0.58 (0.12)	0.42 (0.1) a	0.35 (0.15) ab	0.24 (0.11)	0.24 (0.06)

CTRL—control subjects; PSO—psoriatic patients; suffix_t—tissue; suffix_s—serum; (nl)—non-lesional; (l)—lesional; CER—ceramide; S1P—sphingosine-1-phosphate; SFA—sphinganine; SFA1P—sphinganine-1-phosphate; SFA/SFO—sphinganine/sphingosine ratio; SFO—sphingosine; a—different vs. CTRL (*p* < 0.05); b—different vs. PSO (*p* < 0.05).

## Data Availability

The data presented in this study are available on request from the corresponding author.
